# Effects of maternal dietary omega-3 polyunsaturated fatty acids and methionine during late gestation on fetal growth, DNA methylation, and mRNA relative expression of genes associated with the inflammatory response, lipid metabolism and DNA methylation in placenta and offspring’s liver in sheep

**DOI:** 10.1186/s40104-020-00513-7

**Published:** 2020-11-18

**Authors:** Milca Rosa Velazquez, Fernanda Batistel, Juan Manuel Pinos Rodriguez, Alejandro Enrique Relling

**Affiliations:** 1 Facultad de Medicina Veterinaria y Zootecnia, Universidad Veracruzana, 91710 Veracruz, Mexico; 2 Department of Animal Science, Ohio Agricultural Research and Development Center (OARDC), The Ohio State University, 114 Gerlaugh Hall, 1680 Madison Ave, Wooster, OH 44691 USA; 3 Department of Animal, Dairy and Veterinary Sciences, Utah State University, Logan, UT 84322 USA

**Keywords:** Fat supplementation, Fetal programming, Maternal nutrition, Nutrients, Rumen protected methionine

## Abstract

**Background:**

Omega-3 PUFA or methionine (Met) supply during gestation alters offspring physiology. However, the effect of both nutrients on fetal development has not been explored. Our objective was to determine the effects of supplementation of these two nutrients during late gestation on fetal growth, DNA methylation, and mRNA expression of genes associated with the inflammatory response, and DNA methylation. Ewes (*n* = 5/treatment) were fed from day 100 to 145 of gestation one of the following treatments: 1) basal diet (NS) without fatty acids (FS) or methionine (MS) supplementation; 2) FS (10 g/kg Ca salts, source omega-3 PUFA); 3) MS (1 g/kg rumen protected methionine); and 4) FS and MS (FS-MS). On day 145, ewes were euthanized, and data from dams and fetus was recorded. Placenta (cotyledon), fetal liver, and blood samples were collected.

**Results:**

A treatments interaction on fetal liver weight, ewe body weight and body condition score (BCS) was observed; FS-MS were heavier (*P* <  0.01) than FS and MS, and FS-MS ewes had a better (*P* = 0.02) BCS than NS. Methionine increased (*P* = 0.03) ewe plasma glucose concentration. Fetal liver global DNA methylation increased (*P* <  0.01) in FS and MS. Dietary treatments modify the mRNA relative expression on some of the genes evaluated. In the fetal liver, FS increased (*P* = 0.04) the mRNA relative expression of arachidonate-5-lipoxygenase-activating-protein and tended to decrease (*P* = 0.06) methionine-adenosyltransferase-1A. Moreover, MS decreased (*P* = 0.04) DNA-methyltransferase-1 and tended to decrease (*P* = 0.08) free-fatty-acid-receptor-1 mRNA relative expression. Furthermore, FS-MS decreased mRNA relative expression of tumor-necrosis-factor-alpha (*P* = 0.05), peroxisome-proliferator-activated-receptor-delta (*P* = 0.03) and gamma (*P* = 0.04), tended to decrease (*P* ≤ 0.09) interleukin-6, fatty-acid-transport-protein-1, and delta-5-desaturase, and increased adenosylhomocysteinase (*P* = 0.04) mRNA relative expression. In cotyledon, FS tended to decrease fatty acid binding protein 4 (*P* = 0.09) mRNA relative expression.

**Conclusion:**

Omega-3 PUFA and Met supplementation improves dam’s performance in late gestation, which was positively correlated with an increase in offspring’s liver development. Moreover, FS-MS decreased mRNA relative expression of proinflammatory cytokines, and lipogenic genes, and increased the expression on an enzyme that has an important role in methylation.

## Background

A growing body of evidence suggests that maternal nutrition has fetal programming effects. The fetal programming theory proposes that environmental stimuli, e.g. nutrition, during the fetal developmental phase has life lasting effects [1, 2]. In ruminants, changes in offspring’s growth and physiology after maternal supply of omega-3 polyunsaturated fatty acids (PUFA) or methionine (Met) during late gestation has been associated with alterations in gene expression [3, 4]. Thus, the possible lasting effects in metabolism and performance of the offspring, by altering maternal diet during gestation, could be a tool to enhance animal health and productivity of food producing animals. Moreover, this study could help in the development of a research model that studies the effect of dietary supplementation with both nutrients during gestation on fetal programming in humans, since the pregnant ewe model has been widely used to investigate maternal-fetal nutrient transfer and placental development in humans [5].

Fatty acids are involved in a variety of biological processes and systems, including the immune system [6, 7], enzymatic activities, cellular proliferation and differentiation, receptor expression [8] and regulation of gene expression [9]. Maternal dietary omega-3 PUFA intake has demonstrated beneficial effects on pregnancy outcomes, including increased fetal and placental growth in mice [10]. Omega-3 PUFA are ligands for transcription factors involved in gene regulation of metabolic and developmental processes [9]. Supplementation with omega-3 PUFA have shown changes not only in offspring growth [3], but also changes in the mRNA expression on the fetal part of the placenta [11]. One of the genes affected by the omega-3 PUFA supplementation was the DNA-methyltransferase (DNMT)-3A [11], an enzyme that methylate DNA. Another gene in the cotyledon affected by maternal supply of omega-3 PUFA was free fatty acid receptor (FFAR)-4 [11], a gene involved in lipid metabolism. Similarly, supplementation with omega-3 PUFA has affected the mRNA expression of genes involved in lipid metabolism within the liver, but much of this research has been done in adult animals [12, 13]. Omega-3 EPA (C20:5n-3) and DHA (C22:6n-3) might affect the inflammatory response by inhibiting production of pro-inflammatory cytokines [14, 15]. Also, omega-3 PUFA can be substrate for potent anti-inflammatory and pro-resolving lipid mediators named resolvins [16, 17]. Supplementation with omega-3 PUFA enhanced resolvins basal concentrations [18–20], and increased the mRNA expression of lipoxygenases (*ALOX*), enzymes that synthetized resolvin D1 (RvD1) [19].

Methionine has been associated with many metabolic functions [21–25]. Supplementation with methyl donors during gestation reported contradictory effects in the immune response of the dam and the newborn [21–23]. One study reported a down regulation of genes related to pro-inflammatory response in follicular cells of multiparous Holstein cows [21]. However, an altered expression of genes associated with immunity were observed after folic acid supplementation [22]; but no effect on pro-inflammatory cytokines [23] was observed when Met was supplemented to dairy cows. Dietary Met supply can alter DNA methylation since it depends on the availability of methyl donors supplied, such as Met [24]. Methylation of DNA is a significant mechanism for gene expression regulation; therefore, a mechanism of how fetal programming occurs [24]. Methylation of DNA is carried out by the covalent addition of a methyl group from S-adenosylmethionine (SAM) to cytosine by the action of DNMT enzymes [25]. An increase of methionine-adenosyl-transferase-1A expression (MAT1A), the enzyme that starts the reaction that converts Met to SAM in the Met cycle, was observed after Met supplementation in dairy cows during the peripartal period with a concomitant increase in the gene expression of catalytic DNMT [25].

Studies have investigated the fetal programming effects of maternal supplementation with omega-3 PUFA or Met during gestation and their effect on mRNA expression in ruminants. However, and to our knowledge, no study has explored the effect of both nutrients on fetal growth, mRNA expression of genes involved in the inflammatory response, lipid metabolism, and DNA methylation. It is unknow whether the maternal dietary intake of both omega-3 PUFA and Met during late gestation will improve fetal growth, and development. Differently from previous studies [3, 4, 9, 18, 21, 25], the aim of this study was not only to determine the effects of supplementing omega-3 PUFA, or Met, but to determine the effects of the combination of these two nutrients to ewes during late gestation on fetus growth, global DNA methylation in fetal tissues, and mRNA expression of genes involved in placenta and fetal liver inflammatory response, lipid metabolism and DNA methylation. The placenta is the site where nutrient exchange between the mother and the fetus occurs, thus modifying maternal diet by the addition of nutrients that can change placenta gene expression could have an important role in fetal growth and development [9].

Omega-3 PUFA can modulate the expression of genes associated with the inflammatory response, lipid metabolism, and DNA methylation [8, 9, 11]. On the other hand, supplementation with Met can increase the availability of methyl donors affecting DNA methylation [25], and the expression of genes associated with the inflammatory response [21–23]. Therefore, the hypothesis of the present study is that supplementation with omega-3 PUFA and Met to ewes during the last third of gestation increases fetal growth and DNA methylation in fetal tissues, decreases the mRNA expression of inflammatory cytokines, increases the mRNA concentration of enzymes involved in the Met cycle, DNA methylation, and in the RvD1 metabolic pathway. In addition, we hypothesized that maternal supplementation increases mRNA concentration of lipolytic genes and decreases mRNA concentration of lipogenic genes in both tissues.

## Methods

### Experimental design, animals, and treatments

This research study was conducted at the Sheep Research Center of the Ohio Agricultural Research and Development Center, Wooster, Ohio. All procedures involving animals were approved by The Ohio State University Institutional Animal Care and Use Committee (IACUC #2019A00000001). In 2 × 2 factorial arrangements of treatments 20 multiparous pregnant Dorset × Hampshire ewes (body weight [BW] 96.2 ± 14.6 kg) between 3 to 4 years of age were housed in 20 pens (1 ewe per pen) and supplemented from day 100 to 145 (5 days before the expected parturition day) of gestation. Five Dorset rams were used (1 ram per block of 4 ewes) for natural service. The sire was part of the block error. Day one of pregnancy was considered the day on which a standing estrus was confirmed by the use of rams. On days 45 to 50 of gestation a pregnancy check was performed, and only pregnant ewes were allocated into the experiment. Number of fetuses was recorded on day 145. The main factors were omega-3 PUFA and Met supplementation. Ewes were blocked by age and BW; and within each block randomly assigned to one of four treatments (5 pens per treatment): 1) A basal diet (no supplementation; NS) was fed to meet pregnant ewe nutrient requirements during late gestation [26], 2) Basal diet plus a source omega-3 PUFA (FS; 10.1 g/kg dry matter intake [DMI] with Ca salts of FA [Strata G113, Virtus Nutrition]), 3) Basal diet plus Met (MS; 1.0 g/kg DMI with rumen protected Met [RPM, Smartamine® M, Adisseo]), and 4) Basal diet plus omega-3 PUFA and Met (FS-MS; same doses and sources listed for the FS and MS treatments).

During the experimental period ewes were fed 2.02 kg/d of the basal diet including the different treatments. Table 1 contains the dietary ingredients and chemical composition of the basal diet while Table 2 contains the FA profile of the supplement used. The dose of omega-3 PUFA was based on previous research in pregnant ewes where supplementation at similar doses had effects on lamb growth, and abundances of relevant mRNA transcripts in the adipose tissue of ewe without affecting dam performance [3, 27, 28]. In these two previous studies [27, 28] an intake of at least 18 mg/kg of metabolic BW (BW^0.75^) of EPA and DHA was aimed. The targeted dose was based on research conducted in humans [29] where supplementation with EPA and DHA at this dose improved insulin sensitivity and decreased cardiovascular disease risk. The supplementation used in the current study (StrataG113) contains 16% EPA and DHA (Table 2) and has a biohydrogenation rate of 50% [30]. Of the 1.01% of the Ca salts of fatty acids supplement added to the diet 20.33% correspond to PUFA. A pure commercial source on Ca salts of EPA and DHA is not available. Because there is no data on supplementing RPM to pregnant ewes, the dose of the supplement was determined using previous studies in pregnant cows [31, 32]. Smartamine M, the RPM used, is 75% *DL*-methionine 90% of that methionine pass to the small intestine; and 90% is absorbed. Thus, 60% of this RPM can be found in the bloodstream [33]. Methionine supplementation was individually weighed, added and mixed into the feed daily in a dose of 2.019 g/ewe. A total of 0.1% of methionine supplement was added to the diet. No feed refusals were found during the entire period of the study.

**Table 1:** Ingredients and chemical composition of the basal diet^a^

	**Treatment, g/kg DM**			
	**NS**^**b**^	**FS**^**c**^	**MS**^**d**^	**FS-MS**
Ingredient				
Alfalfa haylage	179.6	179.6	179.6	179.6
Corn silage	305.4	305.4	305.4	305.4
Ground corn	101.0	88.9	101.0	88.9
DDGS	101.0	100.7	101.0	100.7
Limestone	4.4	4.8	4.4	4.8
Soy hulls	306.5	308.8	306.5	308.8
PUFA Ca salts (EPA + DHA)^e^	–	10.1	–	10.1
Rumen protected methionine^f^	–	–	1.0	1.0
Pre-mix minerals and vitamins^g^	2.0	1.8	2.0	1.8
Chemical composition				
Neutral detergent fiber	407.0	417.9	407.0	417.9
Crude protein	154.6	179.9	154.6	179.9
Ash	58.7	64.0	58.7	64.0
Ether extract	36.8	43.7	36.8	43.7

^a^Basal diet containing the different treatments fed to ewes at 2.02 kg/d during the las 50 days of gestation

^b^*NS* Basal diet with no supplementation; dams were fed to meet sheep requirements of nutrients (NRC, 2007)

^c^*FS* fatty acid supplementation; dams were fed to mee sheep requirements of nutrients (NRC, 2007), and supplemented with 10 g/kg of Ca salts of polyunsaturated fatty acids containing EPA and DHA (FA; StrataG113, Virtus Nutrition LLC, Corcoran, CA)

^d^*MS* Methionine supplementation; dams were fed to mee sheep requirements of nutrients (NRC, 2007), and supplemented with 1 g/kg (dry matter basis) of rumen protected methionine (Smartamine® M, Adisseo, Alpharetta, GA)

^e^StrataG113, Virtus Nutrition LLC

^f^Smartamine® M, Adisseo

^g^Vitaferm Concept-Aid Sheep (BioZyme, St. Joseph, MO). Contains 15.5% Ca, 5% P, 16% NaCl, 4% Mg, 2% K, 10 ppm Co, 70 ppm I, 2850 ppm Mn, 16.4 ppm Se, 2500 ppm Zn, 130,000 IU/kg vitamin A, 7500 IU/kg vitamin D_3_, 550 IU/kg vitamin E

*DDGS* Distiller’s dried grains with solubles, *DM* Dry matter, *FS-MS* Supplementation with fatty acids and methionine, *PUFA* Polyunsaturated fatty acids

**Table 2:** Fatty acid profile^a^ of supplement fed to pregnant ewes

**Fatty acid**	**Supplement**^**b, c**^
	**FA, %**
C8:0+C10:0+C12:0	0.12
C14:0	5.99
C16:0	22.01
C16:1	7.40
C18:0	7.47
C18:1 *c*9	17.46
C18:1 other	4.51
C18:2	2.69
C20:0	0.34
C20:1	0.84
C18:3n-3	0.94
C22:0	0.35
C22:1	1.38
C20:3n-3	0.51
C20:4n-6	0.00
C20:5n-3	9.19
C22:6n-3	7.00
Other	12.15

^a^% of total fatty acids of Ca salts containing polyunsaturated fatty acids of supplement fed to pregnant ewes during the last 50 days of gestion

^b^Ca salts of polyunsaturated fatty acids containing EPA and DHA (FA; StrataG113, Virtus Nutrition LLC, Corcoran, CA)

^c^Fatty acid profiles evaluated using the methods of Weiss and Wyatt [36]

### Sampling

Feed samples were taken weekly, pooled and analyzed according to AOAC [34] for dry matter (DM, method number 981.10), crude protein (CP, method number 967.03), and neutral detergent fiber (NDF) and acid detergent fiber (ADF) according to Van Soest et al. [35]. A heat-stable amylase was included in the NDF and expressed including residual ash. Total FA composition of Ca salts was determinate using the methods described by Weiss and Wyatt [36] (Table 2).

Ewes were weighed, blood sampled, and body condition score (BCS) was measured at the beginning of the experiment (day 100 of gestation). Body weight and BCS were recorded once again at 41 day of supplementation. Body condition score was assessed using a 5-point scale [37].

On day 145 of gestation (5 days before expected lambing day, 45 days of supplementation) ewes were euthanized by captive bolt followed by exsanguination; the fetus was removed, and blood (dam and fetus), the entire placenta, and fetal liver (FL) were collected. The approximate length of time from dam euthanasia until sample preservation was within 5 min for the placenta, and 10 min for fetal liver. When there was a set of twins (15 dams) or triplets (2 dams), one fetus was randomly selected from the set, blood sampled, weighed and tissue sampled. There were three dams with singletons.

Blood samples of 10 mL were taken from the jugular vein of ewes (on days 100 and 145 of gestation) and fetuses (45 days of supplementation), transferred into 14 mL polypropylene tubes (VWR International, Radnor, Pa) which contained solutions of disodium EDTA and benzamidine HCl (1.6 mg and 4.7 mg/mL of blood, respectively), and immediately placed on ice for centrifugation. After centrifugation for 25 min at 1800 × *g* and 4 °C, plasma was aliquoted and stored in four individual 1.5 mL micro polypropylene tubes with snap caps (VWR International, Radnor, Pa) at − 80 °C until analysis.

After, the fetus, placenta and liver were weighed; later tissues were collected using sterile scalpel and forceps, placed into labeled cryogenic vials (Thermo Fisher Scientific, Waltham, MA), flash frozen in liquid nitrogen and stored at − 80 °C until further analysis. For the placenta, a placentome was randomly selected, and caruncle (maternal side of the placenta) and cotyledon (fetal side of the placenta) were separated as described previously by Vatnick et al. [38]. Only 19 fetal placenta samples were used for analysis, due to a problem in the separation of the fetal and maternal part in one on the samples (NS).

### Laboratory analysis

Plasma glucose concentrations were determined using a colorimetric commercial assay which uses glucose oxidase and peroxidase (1070 Glucose Trinder, Stanbio Laboratory, Boerne, TX). Plasma NEFA concentration was measured using microtiter plates and a plate reader in a 2-reaction, enzyme-based assay with acyl-CoA synthetase and acyl-CoA oxidase (Wako Pure Chemical 1, FUJIFILM Wako Diagnostics USA Corporation, Richmond, VA) as described previously by Johnson and Peters [39]. Intraassay and interassay coefficients of variations were 3.21% and 2.10% for glucose, and 3.08% and 1.98% for NEFA, respectively.

Fetal placenta RNA extraction was performed using a commercial kit according to the manufacturer’s protocol (D7003T *Quick*-DNA/RNA Miniprep Plus, Zymo research, Agilent Technologies, Tustin, CA). Fetal liver RNA was extracted using the procedure for total RNA Isolation from animal tissue using RNAzol® RT (Molecular Research Center, Inc., Cincinnati, OH) modified for liver tissue. RNAzol® RT (1 mL) and 0.15 g of 0.1 mm Zirconia/Silica beads (Cat. No. 11079101z BioSpec Products, Bartlesville, OK) were added to a 2-mL tube (round bottom safe-lock, cat. No. 022363352 Eppendorf North America), and then chilled on ice. Frozen liver tissues were quickly weighed (0.05 g) into ice cold RNAzol® RT. Sample was homogenized for 1 min with a bead beater at 3450 RPM (MiniBeadBeater-16 Model 607, BioSpec Products, Bartlesville, OK), put on ice for 1 min and then homogenized once again for 1 min. After homogenization manufacturer’s instructions were followed (RNAzol® RT, Molecular Research Center, Inc., Cincinnati, OH). Samples were stored at −80 °C until analysis. Concentration of RNA was measured using UV spectroscopy (Nanodrop Technologies). Sizing, quantitation, integrity, and purity from RNA was assessed using a BioAnalyzer 2100 and RNA NanoChip assay (Agilent Technologies).

Relative mRNA expression was determined using NanoString nCounter XT Assay (NanoString Technologies, Seattle, WA) for 32 genes (Table 3). These genes were chosen based on their involvement in the RvD1 metabolic pathway, inflammation, Met cycle, DNA methylation, FA uptake and release, FA synthesis and transcription factors, and housekeeping genes. This technology is based on direct detection of the targeted molecules using color-coded molecular barcodes, providing a digital simultaneous quantification of the targeted molecules [27]. Total RNA (175 ng) was hybridized overnight with nCounter Reporter (8 μL) probes in hybridization buffer and in excess of nCounter Capture probes (2 μL) at 65 °C for 17 h. After overnight hybridization, probes excess was removed using two-step magnetic beads-based purification on an automated fluidic handling system (nCounter Prep Station). Biotinylated capture probe–bound samples were immobilized and recovered on a streptavidin-coated cartridge. The abundance of specific target molecules was then quantified using the nCounter digital analyzer. Individual fluorescent barcodes and target molecules present in each sample were recorded with a charge-coupled device camera by performing a high-density scan (325 fields of view). Images were processed internally into a digital format and exported as Reporter Code Count (RCC) files [27]. The nSolver Analysis Software 3.0 (Nanostring Technologies, Seattle, WA) was used to analyze nCounter data. Briefly, RCC files were uploaded and all data was normalized to the geometric mean of the housekeeping target genes: *APOB*, *TBP*, *PPIB*, *PGK1*, and *POLR1B* (Table 3). Treatments effect was evaluated for the abundance of mRNA of the housekeeping genes; there were no treatment effects on abundances of any of the five mRNA transcripts of genes of the tissues. Therefore, the five housekeeping target genes were used to normalize the data.

**Table 3:** Gene names and GenBank accession number^a^

**Gene**^**b**^	**Accession number**
Resolvin metabolic pathway	
*ALOX15*	XM_027975516.1
*ALOX15B*	XM_027974935.1
*ALOX5*	XM_015104505.1
*ALOX5AP*	XM_012184625.2
*COX-2*	NM_001009432.1
Inflammatory response	
*TNF*	NM_001024860.1
*IL1B*	NM_001009465.2
*IL6*	NM_001009392.1
Met cycle	
*MAT1A*	XM_012105414.2
*AHCY*	XM_004014507.2
DNA methylation	
*DNMT1*	NM_001009473.1
*DNMT2*	XM_004014246.3
*DNMT3A*	XM_012166008.2
*DNMT3B*	XM_012189044.2
Lipid metabolism	
*DGAT1*	NM_001110164.1
*FATP1*	XM_015095580.1
*FABP4*	NM_001114667.1
*FADS1*	XM_012101996.2
*FADS2*	XM_015103138.1
*ELOVL2*	XM_012101293.2
*FFAR1*	XM_015095580.1
*FFAR4*	XM_015100194.1
*FASN*	NM_001123003.1
*SCD*	NM_001009254.1
*PPARA*	XM_012175774.2
*PPARD*	XM_004018768.3
*PPARG*	NM_001100921.1
Housekeeping	
*APOB*	XM_012175938.1
*TBP*	XM_015097549.1
*PPIB*	XM_004010536.3
*PGK1*	NM_001142516.1
*POLR1B*	XM_004005912.3

^a^Benson et al. [40]

^b^*ALOX15* Arachidonate 15-lipoxygenase, *ALOX15B* Arachidonate 15-lipoxygenase-2, *ALOX5* Arachidonate 5-lipoxygenase, *ALOX5AP* Arachidonate 5-lipoxygenase activating protein, *COX-2* Prostaglandin-endoperoxide synthase 2, *TNF* Tumor necrosis factor-alpha, *IL1B* Interleukin 1 beta, *IL6* Interleukin 6, *MAT1A* Methionine adenosyltransferase 1A, *AHCY* Adenosylhomocysteinase, *DNMT1* DNA methyltransferase 1, *DNMT2* DNA methyltransferase 2, *DNMT3A* DNA methyltransferase 3 alpha, *DNMT3B* DNA methyltransferase 3 beta, *DGAT1* Diacylglycerol O-acyltransferase 1, *FATP1* Fatty acid transport protein 1, *FABP4* Fatty acid binding protein 4, *FADS1* Delta-5-desaturase, *FADS2* Delta-6-desaturase, *ELOVL2* ELOVL fatty acid elongase 2, *FFAR1* Free fatty acid receptor 1, *FFAR4* Free fatty acid receptor 4, *FASN* Fatty acid synthase, *SCD* Stearoyl-CoA desaturase, *PPARA* Peroxisome proliferator activated receptor alpha, *PPARD* Peroxisome proliferator activated receptor delta, *PPARG* Peroxisome proliferator activated receptor gamma, *APOB* Apolipoprotein B, *TBP* TATA-box binding protein, *PPIB* Cyclophilin B, *PGK1* Phosphoglycerate kinase 1, *POLR1B* RNA polymerase I subunit B

The global DNA methylation of placenta and FL was quantified with 5-mC DNA ELISA Kit (D5326, Zymo Research, Tustin, CA) according to the manufacturer’s protocol. This kit uses purified genomic DNA as sample source. Placenta and fetal liver DNA extraction was performed using a commercial kit according to the manufacturer’s protocol (D7003T *Quick*-DNA/RNA Miniprep Plus, Zymo Research, Tustin, CA). The 5-mC DNA ELISA Kit has an anti-5-methylcystosine (5-mC) monoclonal antibody that is both sensitive and specific for 5-mC. The percent 5-mC in the DNA samples was detected and then quantified from a standard curve generated with the designed controls included within this kit. The steps for quantifying the percentage of 5-mC in a DNA sample can be found in the kit manufacturer’s protocol.

### Statistical analysis

The number of experimental units needed per treatment was estimated using previous data, considering mRNA expression of the reported genes variation in fetal programming studies in sheep [4, 27]. All data were analyzed as a randomized complete block design with a 2 × 2 factorial arrangement of treatments, using the MIXED procedure (SAS Institute, Cary, NC) with a model testing the fixed effects of FA supplementation, Met supplementation, and their interaction; and the random effects of ewe within each block and block. Ewe or fetus were considered the experimental unit. Initial BW, and initial BCS, were included as covariables for changes in ewe BW and BCS, respectively. The number of fetus was included as covariables for changes in fetus weight; and fetus weight was included as covariables for placental and FL weight. The covariables were removed when not significant (*P* > 0.10). Least square means (LSMEANS) and standard errors of the mean (SEM) were determined using the LSMEANS statement in the MIXED procedure. The PDIFF option of SAS was used for mean separation when the *P*-value for an interaction was ≤0.10. Significance was set at *P* ≤ 0.05 and tendencies were determined at *P* > 0.05 and *P* ≤ 0.10.

## Results

### Dam performance and plasma concentration of glucose and NEFA

There was an interaction of treatments effect on ewe BW (*P* <  0.01) and BCS (*P* = 0.02). Dams on NS and FS-MS treatments were heavier than those that were supplemented with only FS or MS (Table 4), while ewes supplemented with FS had a greater BCS compared with the ewes in the other three treatments (NS, MS, and FS-MS). No differences (*P* ≥ 0.21) were observed in dam’s plasma NEFA concentration; however, Met supplementation (MS and FS-MS) increased (*P* = 0.03) the plasma glucose concentration compared with the ewes on no Met supplementation treatments (NS and FS) (Table 4).

**Table 4:** Effects of supplementation with omega-3 PUFA and Met on ewe BW, BCS, and plasma metabolites^d^

	**Treatments**				**SEM**	***P*** **values**^**g**^		
	**NS**	**MS**	**FS**	**FS-MS**		**L**	**M**	**L×M**
BW^e^, kg	106.2^a^	99.4^b^	100.2^b^	104.5^a^	1.86	0.81	0.51	< 0.01
BCS^f^	3.2^c^	3.3^b^	3.4^a^	3.3^b^	0.30	0.79	0.96	0.02
Plasma NEFA, μEq/L	466.1	302.9	303.0	319.0	67.89	0.30	0.30	0.21
Plasma glucose, mg/dL	86.1	106.3	81.0	94.7	7.32	0.28	0.03	0.66

^a,b,c^Values with different superscript differ with a *P* value ≤0.05

^d^Data is presented as a least square means ± standard error of the mean (SEM)

^e^Initial BW was used as a covariable

^f^Initial BCS was used as a covariable

^g^L = lipid effect of FA supplementation in the dam diet, M = methionine effect of ME supplementation in the dam, L×M = lipid and methionine effect of FA-ME supplementation in the dam

*BW* Body weight, *BCS* Body condition score, *FS* Fatty acid, *FS-MS* Fatty acids and methionine supplementation, *MS* Methionine supplementation, *Met* Methionine, *NEFA* Non-esterified fatty acids, *NS* Basal diet with no supplementation, *PUFA* Polyunsaturated fatty acids

### Fetus, placenta and fetal liver weight, and fetus plasma concentration of glucose and NEFA

Ewe supplementation did not affect (*P* ≥ 0.16) fetal or placental weight (Table 5). However, there was an interaction of treatment effect (*P* <  0.01) on FL weight; fetuses from dams on NS and the FS-MS treatments had a heavier liver than those from dams who were on the MS or FS treatments (Table 5). There were no differences (*P* ≥ 0.29) on fetus plasma NEFA, nor fetus glucose concentrations (Table 5).

**Table 5:** Effects of supplementation with omega-3 PUFA and Met on fetal, and placental weight, and fetal plasma metabolites^d^

	**Treatments**				**SEM**	***P *values**^**g**^		
	**NS**	**MS**	**FS**	**FS-MS**		**L**	**M**	**L×M**
Placental weight^e^, kg	3.2	2.4	2.7	2.9	0.35	0.98	0.42	0.16
Fetus weight^f^, kg	5.6	5.3	5.1	5.4	0.38	0.67	0.99	0.46
Liver weight^e^, g	122.9^b^	110.7^c^	113.8^c^	126.9^a^	3.70	0.36	0.91	< 0.01
Plasma NEFA, μEq/L	360.1	266.7	237.93	248.4	68.80	0.32	0.56	0.46
Plasma glucose, mg/dL	19.1	12.5	12.6	13.5	3.39	0.43	0.41	0.29

^a,b,c^Values with different superscript differ with a *P* value ≤0.05

^d^Data is presented as a least square means ± standard error of the mean (SEM)

^e^Fetus weight was used as a covariable

^f^Number of fetuses was used as a covariable

^g^L = lipid effect of FA supplementation in the dam diet, M = methionine effect of ME supplementation in the dam, L×M = lipid and methionine effect of FA-ME supplementation in the dam

*FS* Fatty acid, *FS-MS* Fatty acids, and methionine supplementation, *MS* Methionine supplementation, *Met* Methionine, *NEFA* Non-esterified fatty acids, *NS* Basal diet with no supplementation, *PUFA* Polyunsaturated fatty acids

### Placenta and fetal liver DNA global methylation

No treatments effect (*P* ≥ 0.30) was observed in placenta DNA global methylation, and no interactions between treatments (*P* = 0.32) were observed for DNA global methylation of FL. However, there was a main effect of FA and Met supplementation (*P* < 0.01), supplementation with FA or Met increased global methylation in FL. In FL, Met supplementation increased (*P* < 0.01; MS and FS-MS; 57.7% and 58.8% respectively) DNA global methylation when compared with FS and NS (57.4% and 55.8%, respectively) treatments, and at the same time FS increased (*P* < 0.01; 57.4%) DNA global methylation when compared with NS (55.8%) (Supplementary Table 1).

### Cotyledon relative mRNA expression

There was a tendency (*P* = 0.09) for fatty acid binding protein 4 (*FABP4*) mRNA relative expression, where FA supplementation (FS and FS-MS; 15.22 and 20.30 respectively) tended to decrease *FABP4* mRNA relative expression in the cotyledon when compared with NS and MS treatments (31.03 and 22.48, respectively). On the other hand, FS, MS, and FS-MS treatments did not affect (*P* > 0.17) relative expression of the mRNA related to the RvD1 metabolic pathway, the inflammatory response, Met cycle, nor those associated with DNA methylation (Supplementary Table 2).

### Fetal liver relative mRNA expression

In the RvD1 metabolic pathway, dam FA supplementation (FS and FS-MS) increased (*P* = 0.04) arachidonate-5-lipoxygenase-activating-protein (*ALOX5AP*) relative mRNA expression in the FL when compared with NS and MS treatments (Table 6). Among the genes related to the inflammatory response, there was an interaction of treatment effect (*P* = 0.05) on tumor necrosis factor alpha (*TNF*) relative mRNA expression and a tendency (*P* = 0.08) on interleukin-6 (*IL6*) mRNA relative expression. Supplementation with FA and Met decreased *TNF* relative mRNA expression when compared with FS and MS treatments. On the other hand, FS-MS tended to reduce *IL6* relative mRNA expression when compared with the other three treatments. At the same time, MS treatment tended (*P* = 0.10) to increase *IL6* relative mRNA expression in the FL compared with the fetuses from ewes on the other three treatments. Regarding the genes involved in the Met cycle, FA supplementation (FS and FS-MS) tended (*P* = 0.06) to decrease of methionine adenosyltransferase 1A expression (*MAT1A*) relative mRNA expression in the FL when compare with NS and MS treatments (Table 6). There was also a treatment interaction (*P* = 0.04) effect for adenosylhomocysteinase (*AHCY*) relative mRNA expression. Supplementation with FA and Met increased *AHCY* relative mRNA expression in FL compared with FL from ewes on FS, MS, or NS treatments (Table 6). Concerning the genes related to the DNA methylation, Met supplementation (MS and FS-MS) decreased (*P* = 0.04) DNA-methyltransferase-1 (*DNMT1*) relative mRNA expression compared with NS and FS treatments. A FA and Met interaction effect was observed for some of the genes involved in the lipid metabolism (*P* ≤ 0.04). Dam FS-MS treatment had a tendency to decrease fatty-acid-transport-protein-1 (*FATP1*) (*P* = 0.09), and delta-5-desaturase (*FADS1*) (*P* = 0.06) relative mRNA expression, and decreased the relative mRNA expression of peroxisome-proliferator-activated-receptor-delta (*PPARD*) (*P* = 0.03) when compared with fetuses from ewes on NS, FS, and MS treatments. Another treatment interaction effect (*P* = 0.04) was observed, in this case FS-MS treatment decreased peroxisome-proliferator-activated-receptor-gamma (*PPARG*) relative mRNA expression when compared with fetuses from ewes on FS and MS treatments, but the FL relative mRNA expression of *PPARG* of FS-MS increased when compared with NS. Furthermore, Met supplementation (MS and FS-MS) tended to (*P* = 0.08) decrease free-fatty-acid-receptor-1 (*FFAR1*) relative mRNA expression compared with NS and FS treatments.

**Table 6:** Effects of supplementation with omega-3 PUFA and Met on fetal liver mRNA expression^d^

**Gene**	**Treatment**				**SEM**	***P*** **values**^**e**^		
	**NS**	**MS**	**FS**	**FS-MS**		**L**	**M**	**L×M**
Resolvin metabolic pathway								
*ALOX15*	17.94	22.62	17.276	21.71	7.32	0.91	0.50	0.99
*ALOX15B*	13.66	10.24	9.40	8.40	3.28	0.32	0.47	0.69
*ALOX5*	48.19	54.44	53.71	45.68	5.81	0.76	0.88	0.19
*ALOX5AP*	8.98	6.40	10.73	11.75	1.77	0.04	0.63	0.28
*COX-2*	145.51	148.7	138.17	180.01	28.83	0.65	0.40	0.47
Inflammatory response								
*TNF*	13.41^b^	20.44^c^	19.76^c^	12.68^a^	3.61	0.83	0.99	0.05
*IL1B*	51.46	58.71	66.67	60.25	9.73	0.36	0.96	0.45
*IL6*	2.05	2.17	4.78	1.31	1.07	0.35	0.10	0.08
Met cycle								
*MAT1A*	9209.03	8098.25	7150.28	7074.33	864.51	0.06	0.46	0.52
*AHCY*	1904.50^b^	1713.29^c^	1594.18^c^	2543.81^a^	288.24	0.33	0.16	0.04
DNA methylation								
*DNMT1*	199.51	166.00	217.60	140.60	26.97	0.88	0.04	0.38
*DNMT2*	180.94	173.22	159.46	143.92	21.68	0.21	0.56	0.84
*DNMT3A*	256.89	285.27	271.28	246.83	22.96	0.57	0.93	0.22
*DNMT3B*	11.19	10.75	12.63	8.64	1.85	0.85	0.20	0.30
Lipid metabolism								
*DGAT1*	448.25	522.41	529.36	275.58	112.92	0.43	0.39	0.13
*FATP1*	37.09	42.05	41.60	26.46	6.13	0.33	0.37	0.09
*FABP4*	8.10	20.58	3.68	8.66	6.78	0.20	0.17	0.55
*FADS1*	597.45	837.10	813.79	578.39	131.67	0.86	0.99	0.06
*FADS2*	574.01	691.07	671.96	698.91	193.61	0.77	0.69	0.80
*ELOVL2*	13.72	23.08	13.96	15.76	3.71	0.31	0.11	0.28
*FFAR1*	8.89	4.30	11.08	5.81	2.93	0.49	0.08	0.89
*FFAR4*	16.66	12.65	25.89	24.05	6.75	0.11	0.64	0.86
*FASN*	15.88	16.92	17.38	19.04	2.64	0.46	0.58	0.89
*SCD*	331.60	293.74	374.25	294.43	80.28	0.77	0.43	0.78
*PPARA*	1231.39	1246.15	1069.26	1282.38	138.08	0.62	0.37	0.44
*PPARD*	37.09^b^	40.4^c^	40.12^c^	26.38^a^	3.84	0.13	0.15	0.03
*PPARG*	17.63^c^	25.87^a^	27.29^a^	23.65^b^	2.88	0.17	0.39	0.04

^a,b,c^Values with different superscript differ with a *P* value ≤0.05

^d^Data is presented as a least square means ± standard error of the mean (SEM)

^e^L = lipid effect of FA supplementation in the dam diet, M = methionine effect of ME supplementation in the dam, L×M = lipid and methionine effect of FA-ME supplementation in the dam

*FS* Fatty acid, *FS-MS* Fatty acids and methionine supplementation, *MS* Methionine supplementation, *Met* Methionine, *NEFA* Non-esterified fatty acids, *NS* Basal diet with no supplementation, *PUFA* Polyunsaturated fatty acids

## Discussion

The intrauterine environment plays an important role during fetal development; adequate fetal growth is essential and contributes critically to the long-term performance and health of the individual [41]; therefore, dam nutrition during gestation has a key role in the adult performance of the progeny [42]. Supplementation with omega-3 PUFA or Met has beneficial effects on development and growth of the offspring, and supplementation with one or the other has showed to modify the expression of immune mediators, enzymes involved in DNA methylation, and increased growth, but the effects of supplementation with both nutrients on fetus growth, gene expression of cytokines in the NF-κB pathway, enzymes present in the methionine cycle, DNA methylation, and in the RvD1 omega-3 PUFA metabolic pathway, as well as on global DNA methylation of fetal tissues, have not been studied. According to previous studies [4, 11, 28], we expect that fetus growth from dams supplemented with these two nutrients will be associated with an increase in the mRNA expression of the enzymes that participates in DNA methylation (DNMT), Met cycle, and RvD1 metabolic pathway after PUFA supplementation. We assume that PUFA supplementation would modified the expression of DNMT enzymes and Met supplementation will increase methyl donors, thus increasing DNA methylation. Therefore, supplementation with these two nutrients would modify the mRNA expression of inflammatory mediators and genes involved in lipid metabolism; providing the placenta and fetus with a better environment for in utero growth. To our knowledge, this is the first study to report the effects of supplementing both, omega-3 PUFA, and Met during the last third of gestation on dams and fetuses.

### Dam performance, and plasma glucose and NEFA concentration

According to the available literature [3, 27] we were not expecting any differences due to dietary treatments on dam’s BW or BCS. However, ewes in the NS and the FS-MS showed an unexpected increase in BW. Our results differ with the ones reported by Sheibani et al. [43], and two other previous studies [3, 27] where feeding different sources of omega-3 PUFA during the last third of gestation did not show differences in ewe prepartum BW or BCS. Likewise, Waterman et al. [44] and Osorio et al. [45] did not report any differences in BW or BCS when RPM was fed during late gestation. The inconsistent results reported in the current experiment with the ones aforementioned [3, 27, 43–45] might have been related to the interaction between omega-3 PUFA and Met, or to differences in the amount fed, as well as in the type of omega-3 PUFA and Met supplementation, and the length of feeding. We could not establish any physiological explanation for the current findings on dam performance when PUFA and Met were supplemented in late gestation, since no differences in DMI were observed, or any association between ewe’s performance and plasma metabolites of NS or FS-MS was established. Additionally, we did not find any other studies where both omega-3 PUFA and Met were supplemented together during gestation nor the peripartum period, that could help us hypothesized the reason why ewes in the NS and the FS-MS treatments were the heaviest.

To evaluate how the dietary treatments affected the nutritional status of late gestating ewe, plasma metabolites were measured at the initiation and termination of the 45-day trial. In accordance with previous studies where omega-3 PUFA [46] or Met [23] were supplemented during late gestation, we did not expect differences in dam’s plasma NEFA or glucose concentrations. However, we observed an increase in plasma glucose concentration in Met supplemented dams. The difference between our results and the ones reported in previous studies [23, 46] could be due to the longer period Met was supply, and the greater amount of Met supplementation fed in the current study. While Jacometo et al. [23] fed Met at a rate of 0.08% of dry matter for the last 21 days of gestation, we feed it at a rate of 0.10% of dry matter for 45 days at the end of gestation.

### Fetus, placenta and fetal liver weight, and fetus plasma concentration of glucose and NEFA

According to the available literature [10, 28, 47, 48], we expected a greater increase in the fetal, placental, and FL weights of fetus from ewes that were fed both nutrients, despite inconsistencies observed in results after FA supplementation in late gestation [3, 9, 28, 46]. Jones et al. [10] reported an increased in fetal and placental growth after omega-3 PUFA supplementation during late gestation in rats. In accordance with Jones et al. [10], Carranza Martin et al. [28] reported an increase in birth BW in lambs born from dams supplemented with 0.39% Ca salts of n-3 PUFA during the last 50 days of gestation. Likewise, a heavier birth weight was associated with in late gestation RPM supplementation in Holstein cows, suggesting that maternal supply of Met during late pregnancy enhanced in utero growth [47, 48]. However, we did not observe differences in fetal or placental weight, which is in accordance with previous studies [3, 46] where ewe supplementation with omega-3 PUFA during late gestation did not affect lamb BW at birth. Nonetheless, the FL weight of fetuses from ewes supplemented with both omega-3 PUFA and Met were heavier suggesting a greater development of FL; which is in accordance with what was found in others studies conducted in murine, ovine, and bovine models after dam omega-3 PUFA or Met supplementation in late gestation [10, 28, 47, 48]. Moreover, NS and FS-MS fetal liver heavier weight was associated to the increase in dam prepartum BW, suggesting that ewe growth in late gestation could influence fetal liver growth. Additionally, and in order to explain the increase in FL weight it is important to mention that glucose plays a key role in placental and fetal growth as a main energy substrate [48]. However, no changes in fetus plasma metabolites were reported in the current study; therefore, and according to our results we cannot give any physiological explanation to the increase in FL weight observed.

Metabolites were measured in fetal plasma to evaluate how maternal dietary supplementation might affect their concentration in fetal circulation, and consequently affected fetal growth and metabolism. As we were expecting, our results agreed with the ones reported in previous studies [28, 46] where no differences in plasma glucose and NEFA concentrations were observed after dam supplementation during late gestation.

### Cotyledon and fetal liver mRNA relative expression

Omega-3 PUFA are ligands for transcription factors that participate in the regulation of genes involved in the inflammatory response [9]. Resolvins are lipid mediators derived from EPA and DHA [49] by a series of biosynthetic steps involving ALOX and COX enzymes; *ALOX15, ALOX15B* and *COX-2* are involved in the initial step of RvD1 formation, while *ALOX5*, *ALOX5AP* drive the final formation step of these lipid mediators [17]. The basal concentration of these FA derived mediators can be enhanced in rodent liver [18], placenta [19], bone marrow [50] and in human blood [20] by increasing substrate supply via dietary supply of omega-3 PUFA, but there is limited data in sheep. An increase in the relative mRNA expression of *ALOX15B* and *ALOX5* was stimulated in rat placenta by omega-3 PUFA dietary intake in late gestation with a concomitant increase in placenta and fetus weight, suggesting that exposure to dietary omega-3 PUFA could increase the enzymatic conversion to resolving lipid mediators, which may have beneficial effects in the inflammatory response and fetal development [19].

Likewise, maternal dietary supplementation with methyl donors during gestation can modify the expression of genes associated with the inflammatory response in the dam [21] and its progeny [22]. Based on current literature, we were expecting an increase of the relative mRNA expression of genes involved in the anti-inflammatory response. Thus, an increase in relative mRNA expression of the enzymes in the RvD1 metabolic pathway in the cotyledon and fetal liver from ewes fed with both nutrients was expected. Increased capacity of the placenta to resolve inflammation may protect the tissue from detrimental effects of elevated inflammation associated with gestation, which could promote placental and fetal growth [19]. However, we did not find any differences on the mRNA expression of these enzymes in the cotyledon. The differences in our results with the ones found by Jones et al. [19] could be due to the placental structure used to measure the mRNA expression and the omega-3 PUFA rate fed. Jones et al. [19] fed a diet that contained 33.2% of EPA and DHA which is little more than double of what was used in our study. On the other hand, ovine placenta is cotyledonary, with maternal and the fetal sides, while murine placenta is discoidal; and it has the junctional and labyrinth zones. In our study we used the fetal side of the placenta, the cotyledon, while Jones et al. [19] used the placental labyrinth zone. Even though these two structures carry out a similar function, the histological composition of these two placental structures are quite different.

On the other hand, FS increased the mRNA expression of *ALOX5AP* in the fetal liver. Our results are consistent with the ones reported by Gonzalez-Periz et al. [18] where increase hepatic formation of a metabolic precursor for RvD1 was found after omega-3 PUFA supplementation [18].

Partum is considered a state of increased inflammation. Due to this we expected an increase in pro-inflammatory cytokines Tnfα, Il1β, and Il6 at the end of gestation, and according to the available literature, a decrease in the mRNA expression of *TNF*, *IL1B*, and *IL6* in the cotyledon and fetal liver from dams that received FS-MS. However, we did not find any differences in the mRNA expression of these three pro-inflammatory cytokines in the cotyledon, which is not consistent with the well documented anti-inflammatory role of these omega-3 PUFA [14, 51, 52], and with studies where inflammatory cytokines were suppress after Met supplementation [21]. However, Jones et al. [19] reported effects somewhat contradictory on this matter; in their study they found that maternal plasma concentration of TNFα decrease at the end of gestation when dams were supplemented with omega-3 PUFA in late gestation, but maternal plasma concentration of IL1β and placental mRNA expression of *IL1B*, and *IL6* increased with omega-3 PUFA supplementation. These previous data [19, 21] and our own data suggest it is possible that the documented anti-inflammatory effects of omega-3 PUFA and Met in other tissues and situations could not be observed in the placenta due to a possible unique or unusual regulation of inflammation in this tissue at the end of gestation.

Regarding the mRNA relative expression of pro-inflammatory cytokines in the fetal liver, our results are in accordance with the ones reported by Gonzalez-Periz et al. [18] where omega-3 PUFA supplementation inhibited TNFα release by murine hepatic macrophages. The decrease in hepatic mRNA expression of *TNF*, and the tendency in decrease of *IL6* reported in the present study in the FS-MS treatment agrees with the anti-inflammatory effect attributed to omega-3 PUFA by other authors [15, 17, 19], and it is in line with the results reported by Acosta et al. [21] about the potential anti-inflammatory effect of Met supplementation during gestation.

It has been reported that dietary intake of Met increase circulating availability of this methyl donor, which enters 1-carbon metabolism cycle where it is transformed into SAM by the action of MAT1 [53]. S-adenosylmethionine donates a methyl group to cytosine creating methylated CpG patterns in the genome by the action of DNMTs, hence regulating gene expression [54]. Three of these enzymes (DNMT1, DNMT3a, and DNMT3b) catalyze the addition of methylation marks to genomic DNA, while DNMT3L and DNMT2 do not participate in this catalytic activity [4]. Another enzyme that has an important role in methylation is AHCY. It is involved in a multistep process that breaks down Met. This reaction plays an important role in methylation, since it regulates the addition of methyl groups to other compounds [55].

According to the available literature [25, 53–56], we expected a greater increase in the relative abundance of the genes involved in the Met cycle and DNA methylation in the cotyledon and the fetal liver from dams supplemented with FS-MS. However, the interaction of these two nutrients showed no differences in the mRNA relative expression of any of these genes in the cotyledon. A previous study [11] reported a tendency for the increased of *DNMT3A* in ewe caruncle and cotyledon after supplementation with omega-3 PUFA supplementation during gestation. The difference in our results and the ones reported by Roque-Jimenez et al. [11] could be due to the omega-3 PUFA dose used and the time during gestation when this supplementation was fed. Roque-Jimenez et al. [11] administered a dose of 1.61% of Ca salts containing EPA and DHA of DMI during early gestation, while we fed these omega-3 at a lower rate, 1.01% of DMI, during late gestation.

Our cotyledon results also differ from those reported by Batistel et al. [4] where male calves’ placenta showed a greater *AHCY* mRNA abundance when dams received Met supplementation during late gestation. On the other hand, Batistel et al. [4] observed an increased in the mRNA abundance of *MAT1A*, *DNMT3A*, *DNMT3B* in female calves’ placenta. The differences in results in the present study with the ones reported by Batistel et al. [4] could be due to the tissue used to measure the mRNA expression, and the doses of Met supplementation used in the experiment. The placental samples harvested in our study contained only the fetal side of the placentome (cotyledon), while the ones used by Batistel et al. [4] contained similar amounts of fetal and maternal tissue (cotyledon and caruncle).

Data from the current experiment and the one reported by Batistel et al. [4] and Roque-Jimenez et al. [11] suggest that the effect of omega-3 PUFA and Met supplementation during gestation on the mRNA expression of genes related to Met cycle and DNA methylation may depend on the stage of gestation, the supplement is fed, the placental tissue used (maternal, fetal, or both), and on the sex of the offspring.

As aforementioned, an increase in the relative abundance of the genes involved in the Met cycle and DNA methylation was expected in the fetal liver from FS-MS. Surprisingly, there was a tendency from FS to decrease the *MAT1A* relative abundance in the FL. Nonetheless, FS-MS increased *AHCY* relative expression in the FL as expected. Furthermore, Met supplementation decreased *DNMT1* relative mRNA expression. The difference in our DNMT relative mRNA expression results from what we were expecting are in accordance with the ones reported by Osorio et al. [25]. In the study by Osorio et al. [25], the relative mRNA expression of hepatic *DNMT1* was lower; however, an increase of hepatic *MAT1A*, and *DNMT3A* relative mRNA expression was reported after Met supplementation in dairy cows during the peripartal period. We do not have any physiological explanation for the decreased in the mRNA relative expression of *DNMT1*; hence, it is recommended to evaluate the role of dietary Met supplementation in the down regulation of catalytic DNMTs.

Based on previous studies [3, 11, 27] we expected an increase in the expression of genes involved in the transport and metabolism of FA and a decrease of the ones involved in lipogenic activity in tissues obtained from ewes fed omega-3 PUFA (FS and FS-MS). To our knowledge there are no other studies that evaluate the effects of Met supplementation during late gestation on the relative expression of genes involved in lipid metabolism and transport, the existence literature refers to the effects of FA supplementation on the relative expression of these genes.

In our study, the relative mRNA expression of *FABP4* was the only gene involved in the FA metabolism affected in the cotyledon by ewe supplementation during late gestation. Fatty acid binding protein 4 gene product has high affinity for DHA, and its FA transporting role is exclusive for placental tissue [57, 58]; *FABP4* is constantly participating in the transport of DHA and arachidonic acid throughout the placenta [59]. Unexpectedly, PUFA supplementation tended to decrease *FABP4* relative mRNA expression in the cotyledon. This result is contrary to what we were expecting based in studies conducted in humans [58]. The decreased in the mRNA expression of *FABP4* could suggest a decrease in the transportation of DHA in the cotyledon during late gestation when omega-3 PUFA are supplemented.

According to a previous study [11] we were expecting an increase in the mRNA relative expression of *FFAR4* in the cotyledon from omega-3 PUFA supplemented ewes; however, we did not find any differences. The differences found in our results with what was reported by Roque-Jimenez et al. [11] could be due to the gestation stage the FA supplementation was fed, and its dose. Roque-Jimenez et al. [11] fed the omega-3 PUFA supplementation in early gestation at a higher rate than the ones used in the present study, 1.61% and 1.01% of DMI, respectively.

A different pattern from the one observed in the cotyledon was reported in the relative expression of genes involved in lipid metabolism and transport in FL. Maternal supplementation with Met and omega-3 PUFA during late gestation decreased the relative mRNA expression of *PPARG* and *PPARD*, and tended to decrease *FADS1*, *FATP1*, and *FFAR1* relative mRNA expression.

Delta-5 desaturase and delta-6 desaturase play a key role in PUFA synthesis; these two desaturase enzymes are needed to introduce double bonds at the fifth and sixth carbon from the carboxyl end of FAs, respectively, in order to synthesize EPA and DHA from linolenic acid and arachidonic acid [60]. In accordance with our results, Nickles et al. [3] found a decrease tendency in the relative mRNA expression of *FADS1* followed by dam supplementation with 1% Ca salts containing EPA and DHA during late gestation in ewe subcutaneous adipose tissue. Another study conducted in sheep reported a decrease in mRNA relative expression of *FADS1* in intramuscular adipose tissue after a diet with linseed (high in PUFA) was fed to growing lambs [61]. The downregulation of this gene suggests that FA synthesis of EPA and DHA is decreased in the FL of dietary omega-3 PUFA supplementation. The FA profile of the diet might not be the only factor that affects desaturase expression, *PPARs* induction has an important role on the regulation of this *FADS1* [62, 63]*.* In the present study we observed that omega-3 PUFA and Met supplementation modified *PPARD* and *PPARG* mRNA relative expression in the FL. The data reported in the current study suggest what was reported by other authors [62, 63]; changes in *PPARs* mRNA relative expression might influence *FADS1* mRNA expression. A decrease in *FADS1* mRNA relative expression is in accordance with a decrease *PPARD* and *PPARG* relative expression followed by a FS-MS dietary intake during late gestation.

On the other hand, our result on *FATP1* relative mRNA expression differs from the ones reported by Roque-Jimenez et al. [11], where an increase in the relative abundance of this gene was observed after maternal omega-3 PUFA supplementation during early gestation in fetal liver; but are in accordance with what was reported by Nickles et al. [3] when PUFA supplementation was fed in the last third of gestation, even though a Met supplementation was not used in this trial. Desantadina et al. [64] observed *FATP1* in bovine maternal side of placenta in the first two thirds of gestation. The changes observed in *FATP1* relative mRNA expression suggest that *FATP1* action in FA metabolism may be more important in early gestation.

Free fatty acid receptors depend on the availability of FA to be activated and regulate lipid metabolism and placental expression of genes involved in lipid metabolism [65, 66]. Free fatty acid receptor 1 is activated by medium to long chain FA; most strongly activated by EPA [67]. The decreased tendency in the relative mRNA expression of *FFAR1* reported in the current study after Met supplementation (MS and FS-MS) raised questions about the role of the methyl donor in the activity of *FFAR1*. To our knowledge, there is no specific information about the relationship between dietary Met and the genes involved in FA transport and metabolism in cotyledon and FL, thus a specific physiological explanation to this result could not be given.

### Placenta and fetal liver DNA global methylation

Methylation of DNA is a significant mechanism for gene expression regulation [21], which depends on the availability of methyl donors [24]. It has been reported that increasing Met bioavailability is likely to increase entry of this amino acid into the 1-carborn metabolism cycle where it is initially converted into SAM, a fundamental biological methyl donor [68]. Methylation of DNA depends on SAM availability to regulate gene expression. This biological process is carried out by the covalent addition of a methyl group from SAM to cytosine within a Cyst-phosphate-Gua (CpG) region in the DNA by the action of DNMT [25, 69]. It was observed that methyl groups transferred into DNA are ultimately derived from Met, suggesting that dietary intake of Met might alter DNA methylation [70]. Osorio et al. [25] observed an increase in the mRNA expression of *DNMT3A*, a gene associated with DNA methylation after Met supplementation. Furthermore, Roque-Jimenez et al. [11] reported an increase tendency in the relative mRNA abundance of *DNMT3* in placental tissue followed by FA supplementation in early gestation in sheep. Based on these results [11, 25], we were expecting an association between the relative abundance of the genes involved in the Met cycle and DNA methylation with DNA global methylation in placenta and fetal liver from dams that were fed both nutrients. In other words, there would be an associative effect of these two nutrients increasing DNA global methylation in placenta and fetal liver. This increase should be associated with an increase in the relative abundance of mRNA relative expression of genes involved in the Met cycle and DNA methylation. In the present study an interaction between the two nutrients was not observed; also, no treatment effect was observed in placenta DNA global methylation. Nonetheless, the global DNA methylation of the FL was greater in tissues from dams that were fed omega-3 PUFA or Met suggesting what we were expecting; dietary omega-3 PUFA and Met increase the methylation of DNA. However, no association was observed between the increased of global DNA methylation in FL and the mRNA expression of *DNMTS*, and *MAT1A*. Although, an increase in the mRNA relative expression *AHCY* in FL was reported with the increase in global DNA methylation in FL. Our results differ with the ones reported by Osorio et al. [25] who reported an increase in the mRNA relative expression of *MAT1A* and *DNMT3A* associated with an increased in DNA global methylation in the liver of cows supplemented with Met. The differences in our results with the ones reported by Osorio et al. [25] could be due to the dose of Met used and the time the supplementation was administered. Osorio et al. [25] fed the Met supplementation at a lower rate than in the current study, 0.7 g/kg, and 1.0 g/kg of dry matter consumption respectively, and for a longer period of time (from −21 days to birth to 30 days in lactation).

## Conclusions

This study provides evidence that supplementation with Ca salts containing EPA and DHA and methionine (as RPM) during the last third of gestation can improve ewe performance and fetus development. Furthermore, dam supplementation with these two nutrients increased FL weight, decreased relative mRNA expression of pro-inflammatory cytokines as well as the mRNA expression of lipogenic genes, and increased the mRNA expression of AHCY, an enzyme that breakdowns Met which could have an important role in the methylation of DNA. The increase in fetal growth might be due to an increase in ewe performance. Our results provide information on the effects of supplementation of these two nutrients during late gestation on dam’s and offspring metabolism and performance that allow for development of gestational feed programs that could improve health and productivity of these animals. Future research on this area should focus on evaluating the effect of the interaction of these two nutrients, on different stages of gestation, utero/placenta development, and the long term effect of offspring’s productivity and health.

## Supplementary information

**Additional file 1: Supplementary Table 1.** Effects of supplementation with omega-3 PUFA and Met on placenta and fetal liver DNA global methylation.

**Additional file 2: Supplementary Table 2.** Effects of supplementation with omega-3 PUFA and Met on cotyledon mRNA expression.

## Data Availability

The datasets used and analyzed during the current study are available from the corresponding author on reasonable request.

## Abbreviations

5-mC: 5-Methylcystosine
ADF: Acid detergent fiber
ALOX: Lipoxygenase
APOB: Apolipoprotein B
BCS: Body condition score
BW: Body weight
COX: Cyclooxygenase
CP: Crude protein
CpG: Cyst-phosphate-Gua
DM: Dry matter
DMI: Dry matter intake
DNMN: DNA-methyltransferases
FA: Fatty acid
FS: Fatty acids supplementation
FS-MS: Fatty acids and methionine supplementation
FL: Fetal liver
FI: Fetal intestine
IACUC: Institutional Animal Care and Use Committee
LSMEANS: Least square means
MAT1A: Methionine adenosyltransferase 1A
Met: Methionine
MS: Methionine supplementation
n-3: Omega-3
NDF: Neutral detergent fiber
NF-κB: Nuclear factor kappa-light-chain-enhancer of activated B cells
NS: Basal diet with no supplementation
PGK1: Phosphoglycerate kinase 1
POLR1B: RNA polymerase I subunit B
PPIB: Cyclophilin B
RCC: Reporter Code Count
RPM: Rumen protected methionine
RvD1: Resolvins
SAM: S-adenosylmethionine
SEM: Standard error of the mean
TBP: TATA-Box binding protein
