# Supplementary material for: Effects of maternal dietary omega-3 polyunsaturated fatty acids and methionine during late gestation on fetal growth, DNA methylation, and mRNA relative expression of genes associated with the inflammatory response, lipid metabolism and DNA methylation in placenta and offspring’s liver in sheep
Source: J Anim Sci Biotechnol. 2020 Nov 18;11:111. doi: 10.1186/s40104-020-00513-7 (PMC7672917; doi:10.1186/s40104-020-00513-7)
Supplement: Supplementary file 1 — Additional file 1: Supplementary Table 1. Effects of supplementation with omega-3 PUFA and Met on placenta and fetal liver DNA global methylation. [file 40104_2020_513_MOESM1_ESM.docx]

**Supplementary table 1.** Effects of supplementation with omega-3 PUFA and Met on placenta and fetal liver DNA global methylation^1^

|  | Treatments | | | | SEM | *P*-values^2^ | | |
| --- | --- | --- | --- | --- | --- | --- | --- | --- |
|  | NS | MS | FS | FS-MS |  | L | M | L×M |
| Fetus liver global DNA methylation, % | 55.8^c^ | 57.7^b^ | 57.4^b^ | 58.8^a^ | 0.22 | <0.01 | <0.01 | 0.32 |
| Placenta global DNA methylation, % | 2.63 | 0.63 | 1.99 | 1.30 | 1.23 | 0.99 | 0.30 | 0.61 |
| ^1^Data is presented as a least square means ± standard error of the mean (SEM)  ^2^L= lipid effect of FA supplementation in the dam diet, M= methionine effect of ME supplementation in the dam, L×M= lipid and methionine effect of FA-ME supplementation in the dam.  ^a,b,c^ Values with different superscript differ with a *P* value ≤ 0.05.  Abbreviations used: FS, fatty acid; FS-MS, fatty acids, and methionine supplementation; MS, methionine supplementation; Met, methionine; NEFA, non-esterified fatty acids; NS, basal diet with no supplementation; PUFA, polyunsaturated fatty acids. | | | | | | | | |
