# Supplementary material for: Effects of maternal dietary omega-3 polyunsaturated fatty acids and methionine during late gestation on fetal growth, DNA methylation, and mRNA relative expression of genes associated with the inflammatory response, lipid metabolism and DNA methylation in placenta and offspring’s liver in sheep
Source: J Anim Sci Biotechnol. 2020 Nov 18;11:111. doi: 10.1186/s40104-020-00513-7 (PMC7672917; doi:10.1186/s40104-020-00513-7)
Supplement: Supplementary file 2 — Additional file 2: Supplementary Table 2. Effects of supplementation with omega-3 PUFA and Met on cotyledon mRNA expression. [file 40104_2020_513_MOESM2_ESM.docx]

**Supplementary table 2.** Effects of supplementation with omega-3 PUFA and Met on cotyledon mRNA expression**^1^**

| Gene | Treatment | | | | SEM | *P-*value^2^ | | |
| --- | --- | --- | --- | --- | --- | --- | --- | --- |
|  | NS | MS | FS | FS-MS |  | L | M | L×M |
| Resolvin metabolic pathway | | | |  |  |  |  |  |
| *ALOX15* | 102.68 | 6.73 | 13.70 | 57.14 | 63.25 | 0.75 | 0.66 | 0.25 |
| *ALOX15B* | 27.76 | 34.92 | 29.74 | 30.25 | 6.02 | 0.81 | 0.50 | 0.56 |
| *ALOX5* | 143.34 | 30.86 | 55.66 | 55.14 | 73.87 | 0.65 | 0.42 | 0.42 |
| *ALOX5AB* | 52.68 | 16.19 | 41.09 | 33.48 | 26.69 | 0.91 | 0.38 | 0.57 |
| *COX-2* | 29907.00 | 38235.00 | 32023.00 | 40191.00 | 10519.00 | 0.84 | 0.41 | 0.99 |
| Inflammatory response | | |  |  |  |  |  |  |
| *TNF* | 11.61 | 15.04 | 16.01 | 15.88 | 3.55 | 0.44 | 0.62 | 0.59 |
| *IL1B* | 129.50 | 86.18 | 75.85 | 91.81 | 23.59 | 0.29 | 0.54 | 0.19 |
| *IL6* | 51.84 | 33.14 | 174.34 | 184.55 | 123.40 | 0.25 | 0.97 | 0.90 |
| *Met cycle* |  |  |  |  |  |  |  |  |
| *MAT1A* | 4.34 | 7.23 | 6.48 | 50.13 | 24.61 | 0.34 | 0.32 | 0.38 |
| *AHCY* | 1405.13 | 1351.91 | 1368.19 | 1291.41 | 180.75 | 0.77 | 0.70 | 0.94 |
| DNA methylation | |  |  |  |  |  |  |  |
| *DNMT1* | 294.57 | 254.32 | 282.61 | 319.41 | 73.95 | 0.70 | 0.98 | 0.58 |
| *DNMT2* | 505.30 | 426.85 | 443.78 | 459.49 | 82.05 | 0.85 | 0.68 | 0.54 |
| *DNMT3A* | 126.02 | 134.22 | 125.49 | 125.41 | 18.50 | 0.79 | 0.82 | 0.81 |
| *DNMT3B* | 36.45 | 30.41 | 27.95 | 28.44 | 3.86 | 0.19 | 0.48 | 0.41 |
| Lipid metabolism | |  |  |  |  |  |  |  |
| *DGAT1* | 567.71 | 468.26 | 505.40 | 486.02 | 39.62 | 0.55 | 0.12 | 0.29 |
| *FATP1* | 64.46 | 72.03 | 58.74 | 50.80 | 9.68 | 0.15 | 0.98 | 0.39 |
| *FABP4* | 31.03 | 22.48 | 15.22 | 20.30 | 5.31 | 0.09 | 0.73 | 0.18 |
| *FADS1* | 1662.86 | 2117.20 | 1840.03 | 1970.76 | 274.61 | 0.95 | 0.27 | 0.53 |
| *FADS2* | 1760.14 | 2154.72 | 2289.82 | 1890.08 | 316.80 | 0.66 | 0.99 | 0.19 |
| *ELOVL2* | 101.35 | 96.99 | 278.57 | 519.07 | 254.22 | 0.22 | 0.62 | 0.60 |
| *FFAR1* | 8.55 | 8.56 | 9.73 | 10.97 | 2.99 | 0.52 | 0.82 | 0.83 |
| *FFAR4* | 5.12 | 3.39 | 122.48 | 37.58 | 68.71 | 0.25 | 0.50 | 0.52 |
| *FASN* | 120.19 | 134.54 | 84.94 | 118.02 | 22.73 | 0.24 | 0.28 | 0.66 |
| *SCD* | 8017.88 | 6621.35 | 5954.82 | 5026.70 | 1247.25 | 0.13 | 0.33 | 0.84 |
| *PPARA* | 781.19 | 750.74 | 716.72 | 672.85 | 57.86 | 0.20 | 0.49 | 0.90 |
| *PPARD* | 212.94 | 289.95 | 276.16 | 233.15 | 45.29 | 0.94 | 0.69 | 0.17 |
| *PPARG* | 2861.00 | 3515.72 | 2742.27 | 2729.51 | 652.47 | 0.46 | 0.60 | 0.59 |
| ^1^Data is presented as a least square means ± standard error of the mean (SEM).  ^2^L= lipid effect of FA supplementation in the dam diet, M= methionine effect of ME supplementation in the dam, L×M= lipid and methionine effect of FA-ME supplementation in the dam.  Abbreviations used: FS, fatty acid; FS-MS, fatty acids and methionine supplementation; MS, methionine supplementation; Met, methionine; NEFA, non-esterified fatty acids; NS, basal diet with no supplementation; PUFA, polyunsaturated fatty acids. | | | | | | | | |
